# The effect of training load stress on salivary cortisol concentrations, health parameters and hematological parameters in horses

**DOI:** 10.1016/j.heliyon.2023.e19037

**Published:** 2023-08-09

**Authors:** Martin Massányi, Marko Halo, Eva Mlyneková, Eva Kováčiková, Katarína Tokárová, Agnieszka Greń, Peter Massányi, Marko Halo

**Affiliations:** a AgroBioTech Research Centre, Slovak University of Agriculture in Nitra, Slovak Republic; b Institute of Applied Biology, Faculty of Biotechnology and Food Sciences, Slovak University of Agriculture, Nitra, Slovak Republic; c Institute of Animal Husbandry, Faculty of Agrobiology and Food Resources, Slovak University of Agriculture, Nitra, Slovak Republic; d Institute of Biology, Faculty of Exact and Natural Sciences, Pedagogical University of Cracow, Cracow, Poland

**Keywords:** Training, Horse, Stress, Load, Cortisol, Hematology, Health, Exercise

## Abstract

The performance of sport horses is conditioned not only by the quality of its gene pool, but also by a large number of external factors. The most dominant being nutrition, quality of breeding, level of zootechnical care and the quality of the sports rider and coach. Important factor is the process of individuals’ adaptation to the training load occurring during the training itself. This study was focused on the analysis of salivary cortisol levels as well as hematological and biochemical blood parameters in relation to load to which the tested horses were subjected. In the study 14 horses of sport breeds were analyzed a all tested horses were in the same (medium) level of training load. Tested horses underwent following stages of workload – transportation, jumping training, parkour competition, treadmill training, riding training, shoeing and lunging of various intensity. Saliva samples were obtained using a tampon on a string which was inserted into horse's oral cavity, chewed by the horse and placed in a sterile tube with a closable lid. Afterwards, the samples were then stored in deep-freezing boxes at temperature of −80 °C. The EIA cortisol kit was used in this study. The absorbance was read at the wavelength of 450 nm against a reference wavelength of 620–630 nm or a blank sample. Blood samples were obtained at the beginning of the experiment, after half a year of running the experiment and at the end of the experiment from *v. jugularis*. Hematological analysis were carried out using automatic hematologic analyser and multiple parameters were observed. Analysis of biochemical parameters in blood serum were realized using commercial DiaSys kits and semiautomatic biochemical spectrophotometer. Sodium, potassium and chlorides were measured using automatic analyzer EasyLytePlus. In all monitored forms of exercise (transportation, jumping training, parkour competition, treadmill training, riding training, horse shoeing, lunging), an increase in cortisol concentrations immediately after the exercise was recorded, but only spotted statistically significant differences were found during the transportation of monitored horses. The levels of blood parameters were within the reference range during the experiment period. From a comprehensive evaluation of the results, it can be stated that there were no visible health changes to the horses that underwent the experimental load and that manipulation with horses is an important factor that has effect on horses’ stress response. In general, the results of this study show no visible impact of training and/or load on the health status of horses over entire 12-month duration of the experiment.

## 1 Introduction

Horse breeding was, mostly in horses bred for their muscular work, evaluated mainly by their exterior. Even at first glance, it is obvious that good horses in draft, jumping or racing have different body shapes suitable for maximum performance in individual discipline [1]. The performance of a sport and/or racing horse is conditioned by gene pool, as well as by anaerobic metabolism and effective heat dissipation capability as well as by large number of external factors. Most dominant of these are nutrition, quality of breeding, zootechnical care level and finally the quality of the rider and coach [2,3]. Knowledge on stress coping styles can provide valuable information to predict the behavior of individuals during response to specific challenging situations. Moreover, assessing individual differences in adaptation strategies can be useful in horse selection to different exploitation tasks and reproduction [4]. An important factor is the process of adaptation to the work/training load. The first step to intensify and improve the training process is knowing horse physiology. The correct composition of the training load induces a whole complex of positive changes in trained individuals. Limits of load must be set to maintain all animal health requirements and high-performance levels at the same time [5].

In deciding the values of stress factors in horses it has been stated that they can be determined from samples of blood, saliva, or feces. For horses, taking blood samples induce tension. Other strategies that may be included among the non-invasive alternative methods used to monitor acute stress in horses have also been reported. These strategies include changes in the amounts of blinks in the eye and twitches in the eyelid [6]. Also infrared thermography technique, serum cortisol, blood count, and hearth rate (HR) are used as stress-indicators before and after training of different intensities [7]. While long-term transportation affects the immune function in horses, the extent to short-term transport and/or stress impacts remains unclear, although similar physiological and endocrine factors are affected [8]. Also, the analysis of total lactate dehydrogenase (LDH) activity and predominantly its isoenzyme (LDH-4, LDH-5) pattern can significantly contribute to the diagnosis of diseases, which are linked to tissue damage [9].

Stress is a set of non-specific regulatory responses that are engaged when the internal homeostasis in endangered. Stress response is triggered by the activation of some parts of the nervous and endocrine systems. It causes the mobilization of nutrient stores and their transport to tissues with a preferential supply with the ultimate goal being the adjustment of fluctuating values of the body's internal environment [[8], [9], [10], [11], [12]]. Stress response can be divided into three phases: alarm response, adaptation phase and the depletion phase [12].

During the alarm response, catecholamines are immediately secreted which causes increase in blood pressure, glycogenolysis (blood sugar increase) and lipolysis which provides metabolic substrates for muscle work. At the same time, it activates the CRH-ACTH-cortisol system and increases the secretion of cortisol. Subsequently, during the adaptation phase, further activation of the POMC and CRH-ACTH-cortisol system occurs [11]. Cortisol has gluconeogenic and lipolytic (release of fatty acids and glycerol) effects – it provides a substrate for energy reactions. The ability to withstands stress is the highest in this phase [12]. After this phase, the phase of depletion occurs – prolonged or excessive exposure causes damage to the adrenal cortex and impaired cortisol secretion. The body undergoes so much stress that it can lead to shock, lowering of blood pressure or even a heart failure [8,12,13].

Cortisol is a hormone that belongs to the group of glucocorticoids and is produced by the adrenal cortex. It increases the concentration of glucose in the blood by stimulation of muscle protein breakdown into amino acids taken up by the liver out of the bloodstream [14,15] and supports the fatty acid metabolism more than the metabolism of saccharides which saves glucose for the brain, therefore provides comprehensive regulation of energy metabolism [16]. Significant effect of cortisol is the suppression of inflammatory reactions through mediators of inflammation. On the other hand, it has an effect on fibroblasts in the skin – it slows down the process of wound healing. The main regulator of glucocorticoid secretion is ACTH [15]. Salivary cortisol, a measure of free cortisol, follows the circadian rhythm of serum cortisol. Therefore, saliva sampling is a valid and non-invasive technique useful in chronomedicine to estimate free cortisol [17,18]. Also, the correlation found between cortisol levels and Il-1Ra, WBC and erythrocytes indices open new scenario on the positive role of this hormone on the complex and dynamic physiological adaptation to exercise implemented by the organism to re-establish the homeostatic equilibrium, and, interestingly, to maintain an adequate anti-inflammatory environment after exercise [19].

The aim of this study was to analyze the salivary cortisol concentration in horses related to conditions and loads as transportation, jumping training, parkour competition, treadmill and riding training, shoeing and lunging. Also, the health status (blood parameters) during the study period was analyzed during the experimental period.

## 2 Materials and methods

### 2.1 Biologic material

In the experiment samples obtained from 14 horses of sport breeds – 4 Slovak Warmbloods, 9 Holstein horses and 1 Czech Warmblood were analyzed. Sexes in the sample were represented by 2 mares, 3 stallions and 9 geldings over the duration of 1 year. The tested horses weighed 550–600 kg, were in the age of 5 ± 2 years and were housed in the Experimental Center at Institute of Animal Husbandry (Slovak University of Agriculture, Nitra, Slovak Republic) in box housing with sawdust litter. Animals were fed thrice a day with a complete feed mixture balanced for individual animals’ weight and medium workload. Feed rations were formulated individually according to daily requirements (NRC 2007) from crimped barley and oats (at a ratio of 1:1, 0.6 kg per 100 kg of body weight), meadow hay (1.5 kg per 100 kg of body weight), and supplemental feed mixture in muesli form (0.3 kg per 100 kg of body weight) as also previously reported [20]. Nutritional values of basic feed rations were – digestible energy 22.22 MJ/kg of dry matter, crude protein content 295.2 g/kg of dry matter [21]. All tested horses were in the same (medium) level of training load a year before the experiment and were under usual training process and veterinary health control [22].

### 2.2 Types of experimental workload a saliva sampling

Tested horses underwent following stages of workload:

a. Transportation – group consisted of horses which were loaded into the same transporter in the groups of two; exact the same route was set with the duration of 60 min (58 km) and was repeated twice a week; saliva samples were collected thrice: before loading the horses into transporter; immediately after completing the route; 30 min after unloading from the transporter.
b. Jumping training – the load was always executed by the same pair of horse and rider; training lasted for 60 min; saliva samples were collected three times: before jumping training; immediately after jumping training; 30 min after jumping training.
c. Parkour competition – the experimental group consisted of experienced horses, but also of horses which were racing for their first season; average time the horses spent in the load continuously during the competition day was 60 min; saliva samples were collected during sports events; saliva samples were collected thrice: before the race; immediately after the race; 30 min after the race.
d. Treadmill training – the load of tested horses was realized twice a week on training mechanics regulator HorseGym 2000 (HorseGym 2000 GmbH, Harburg-Grossheim, Germany). Horses had exactly set training load: 10 min of step on straight at 6.7 km h^−1^; 10 min of step uphill (3°) at 6.7 km h^−1^; 5 min of step uphill (6°) at 6.7 km h^−1^ and 15 min of step on straight at 6.7 km h^−1^. The samples were obtained thrice – before the treadmill training; immediately after the training and 30 min after treadmill training.
e. Riding training – tested horses were observed in the process of rider training in medium load level. Training consisted of casual exercise in adequate load levels for the duration of 60 min and was realized twice a week. Saliva samples were obtained thrice – before the riding, immediately after the riding and 30 min after completion of the training.
f. Horse shoeing – is one of the forms of load that horses have to undergo to maintain their health and further use. Shoeing of the horses was realized by certified farrier in the stabling area. Entire process took approx. 50 min and was repeated every 6 weeks. Samples of saliva were collected thrice – before the shoeing, immediately upon completion and 30 min after the intervention.
g. Lunging – another part of training process in horses is lunging. Horse with lunging harness is moving in circle with 12-m diameter and is controlled by coach by lunge and a supporting tool (whip). The lunging training took 30 min and horse exercised in step and trot with regular changes of movement direction. The saliva samples were obtained before the lunging, immediately after the training and 30 min after the lunging.

### 2.3 Collection of saliva samples

Saliva samples were collected at the same time (3:00 p.m.) to avoid changes caused by horses’ circadian cycle. The samples were obtained using a tampon on a string which was inserted into horse's oral cavity using sterile gloves, chewed by the horse and placed in a sterile tube with a closable lid. Afterwards, samples were transported to the laboratory, centrifuged and pipetted into Eppendorf microtubes. The samples were stored in deep-freezing boxes at −80 °C.

### 2.4 Cortisol analysis

The EIA cortisol kit a colorimetric competitive enzyme immunoassay kit with absorbance read at 405 nm was used in this study. In our experiment, horse specific ELISA kit (DIALAB Produktion und Vertrieb von chemisch – Technischen Prudukten und Laborinstrumenten Gesellschaft m. b. H., Wiener Neudorf, Austria) was used. Cortisol (antigen) in the sample competes with horseradish peroxidase (enzyme-labeled antigen) for binding to a limited number of anti-cortisol (antibody) sites in the microplate (solid phase). After incubation, the bound and free fractions were separated by a simple solid phase wash. The HRP (horseradish peroxidase) enzyme in bound fraction reacts with the substrate (H_2_O_2_) and TMB substrate (3,3′, 5,5′- tetramethylbenzidine) to form a blue colour which changes to yellow upon addition of STOP solution (H_2_SO_4_). The intensity of the colour is indirectly proportional to the concentration of the cortisol in sample. The absorbance was read at the wavelength of 450 nm against a reference wavelength of 620–630 nm or a blank sample. The lowest detectable concentration of cortisol is 2.42 ng ml^−1^ with confidence ratio of 95% [20,21]. All samples were analyzed twice to avoid deviations.

### 2.5 Blood collection

Blood samples were obtained at the beginning of the experiment, after half a year of running the experiment (in the middle) and at the end of the experiment from *vena jugularis*. The blood was collected into Hemos H-02 tubes and then transferred into Eppendorf tubes containing EDTA as an anticoagulant or into tubes without coagulant (blood serum). Samples were subsequently used for analysis [19].

### 2.6 Hematological analysis

Hematological analysis were carried out using automatic hematologic analyser Abacus Junior Vet5 (Diatron Mi LDT, Budapest, Hungary) and multiple parameters were observed: WBC (total count of leukocytes), LYM (total count of lymphocytes), MID (mid-size population of monocytes, basophils, eosinophils, blasts, and other immature cells), GRA (total count of granulocytes), LYM% (percentage of lymphocytes), MID% (percentage of mid-size population of monocytes, basophils, eosinophils, blasts, and other immature cells), GRA% (percentage of granulocytes), RBC (total count of erythrocytes), HGB (hemoglobin), HCT (hematocrit), MCV (average volume of erythrocytes), MCH (mean corpuscular hemoglobin), MCHC (mean corpuscular haemoglobin concentration), RDWc (red cell distribution width), PLT (total count of platelets), PCT (percentage of platelets), MPV (average volume of platelets) and PDWc (platelet distribution width).

### 2.7 Analysis of biochemical parameters and ions

Analysis of biochemical parameters in blood serum were realized using commercial DiaSys kits and semiautomatic biochemical spectrophotometer RxMonza (Randox Laboratories, Ltd., UK). Sodium, potassium and chlorides were measured using automatic analyzer EasyLytePlus (The Hague, Netherlands). In our study we observed variety of multiple parameters including mineral profile, energy profile, nitrogen profile and hepatic profile.

### 2.8 Statistical analysis

For statistical analysis, we used the GraphPad Prism 6.1 (6.1 version for Windows, GraphPad Software, La Jolla California USA (www.graphpad.com), program. Values were compared using ordinary one-way ANOVA method, according to sample collections and the level of significance was set to: p < 0,5; p < 0.01; p < 0.001; p < 0.0001 and column statistics were calculated.

## 3 Results

Results of the cortisol concentration related to different load stress and summarized in Table 1.

**Table 1:** Cortisol concentrations during the experiment.

Parameter	Before the load	After the load	30 min after the load	p-value
Transportation				
Cortisol (ng.ml^−1^)	3.24 ± 0.65^A^	4.16 ± 1.24	4.54 ± 1.30^A^	**^A^
Jumping Training				
Cortisol (ng.ml^−1^)	3.53 ± 0.41	3.63 ± 0.44	3.78 ± 0.55	ns
Sports competition				
Cortisol (ng.ml^−1^)	3.60 ± 0.42	4.08 ± 0.86	3.53 ± 0.69	ns
Lunging				
Cortisol (ng.ml^−1^)	3.54 ± 0.32	3.59 ± 0.40	3.66 ± 0.37	ns
Shoeing				
Cortisol (ng.ml^−1^)	3.67 ± 0.50	3.78 ± 0.48	3.61 ± 0.41	ns
Riding training				
Cortisol (ng.ml^−1^)	3.86 ± 0.63	4.09 ± 0.37	3.85 ± 0.49	ns
Treadmill training				
Cortisol (ng.ml^−1^)	3.62 ± 0.45	3.73 ± 0.50	3.62 ± 0.60	ns

Within the same row, means with same letters differ significantly (**P < 0.01); P > 0.05.

### 3.1 Horse transportation

In the experiment, the horses were subjected to transport loads in a special horse transporter. During the transport, each animal completed a route of 58 km, which lasted 60 min. The cortisol values before the loading into the transported were 3.24 ± 0.65 ng ml^−1^, followed by sampling directly after unloading from the transporter, at which the cortisol concentration reached 4.16 ± 1.24 ng ml^−1^. After 30 min of unloading from the transporter, the concentrations of cortisol in horse saliva were 4.54 ± 1.30 ng ml^−1^. A significant (p < 0.05) increase in cortisol concentration 30 min after transport compared to the values taken before transport was detected. Also, an increasing trend in concentrations after transport compared to the value at rest was observed indicating the stress caused by transport (Table 1).

### 3.2 Jumping training

Another part of the training of tested horses was the load in form of jumping which was always performed by the same rider-horse pair as when riding. The training lasted 60 min. Cortisol concentrations in the saliva of horses before training were 3.53 ± 0.41 ng ml^−1^. After the training, the values shown increasing tendency to 3.63 ± 0.44 ng ml^−1^ and half hour after training the measured values reached 3.78 ± 0.55 ng ml^−1^ (Table 1). The changes in the measured values of the tested horses were lower than the values obtained in the samples from normal riding sessions and were not statistically significant.

### 3.3 Sports competition

Horses stabled in the study took part in regional but also international horse-riding competitions. Saliva samples were collected during the days of the competition in which the tested horses compete. The experimental group consisted of animals that had experience with the competition as well as horses that raced for the first season. Average time, that the horses were subjected to the load was 60 min. Cortisol concentrations in saliva before the competition were 3.60 ± 0.42 ng ml^−1^. In samples obtained immediately after competition, the cortisol concentrations changed to 4.08 ± 0.86 ng ml^−1^. Samples obtained 30 min after the competition shown decreasing tendency to 3.53 ± 0.69 ng ml^−1^ (Table 1). The concentrations of cortisol were on the similar level as during normal training day.

### 3.4 Horse lunging

Another load that horses were subjected was lunging. Horses were lunged in the Experimental Centre of Institute of Animal Husbandry, SUA Nitra for the duration of 30 min. During lunging, the cortisol concentrations have barely changed, therefore we presume that this type of training is not stressful for the horses. Values at rest were 3.54 ± 0.32 ng ml^−1^, directly after lunging, measured cortisol concentrations were 3.59 ± 0.40 ng ml^−1^ and reached 3.66 ± 0.37 ng ml^−1^ 30 min after the training (Table 1).

### 3.5 Horse shoeing

Shoeing of the horses was one of the loads selected for the experiment. Experimental horses were subjected to shoeing in 6-week intervals during the year by same farriers in the Experimental Centre of Department of Animal Husbandry, SUA Nitra. Shoeing process took different amounts of time in individual horses with average time being 50 min. Cortisol concentrations before the shoeing were 3.67 ± 0.50 ng ml^−1^, 3.78 ± 0.48 ng ml^−1^ directly after shoeing and 3.61 ± 0.41 ng ml^−1^ 30 min after the load (Table 1).

### 3.6 Riding training

The experimental riding load was always performed by the same horse-rider pair in the experimental center of the Institute of Animal Husbandry, and the load itself lasted for 60 min. The concentrations of cortisol in the saliva of the observed animals reached 3.86 ± 0.63 ng ml^−1^ before the load itself. After completion of the training, an increasing trend was found and the concentrations reached 4.09 ± 0.37 ng ml^−1^ (Table 1). 30 min after the end of the training, the value dropped to an almost identical value with the concentration before the training (3.85 ± 0.49 ng ml^−1^).

### 3.7 Treadmill training

**Fig. 1:**
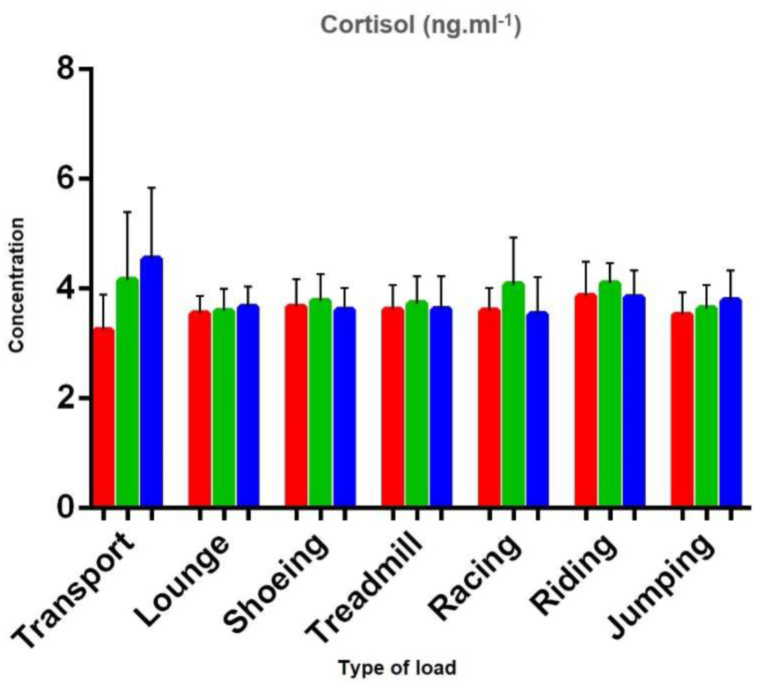
Cortisol concentrations in different load and time periods (before the load – red; after the load – green; 30 min after the load – blue).

In the next stage of our experiment, we took saliva samples from horses that were exposed to the load on the HorseGym 2000 regulator (Table 1; Fig. 1). During the experimental load, the horses walked on the load regulator for 10 min in step on the straight with a speed of 6.7 km h^−1^; 10 min – step uphill (3°) with a speed of 6.7 km h^−1^; 5 min – uphill step (6°) with a speed of 6.7 km h^−1^; 15 min – step on the straight with a speed of 6.7 km h^−1^. Cortisol concentrations in the saliva of horses before exercise reached 3.62 ± 0.45 ng ml^−1^. After training, they shown a rising trend up to 3.73 ± 0.50 ng ml^−1^. However, 30 min after the end of the training on the belt, the values returned to identical to the value before the load itself (3.62 ± 0.60 ng ml^−1^).

From the observed values of cortisol concentrations, which were monitored before the load, directly after the load and 30 min after the end of the load, we can state that all selected loads did not have a significant effect on changes in cortisol concentrations (Fig. 1). Measurements after 30 min of the loads shown decrease of cortisol concentrations back to normal values, except transportation, jumping training and lunge. Statistically significant changes were only found in the samples gathered while transporting horses.

### 3.8 Hematologic parameters

During the experiment also hematological parameters in experimental horses were analyzed to confirm health status. Total count of leukocytes, granulocytes, lymphocytes, hematocrit, total count of thrombocytes etc. were observed before and after the season. All measured values were within reference ranges and there were no visible health changes in horses throughout entire duration of experiment (Table 2). Significant difference within reference range were detected for leucocytes and platelets.

**Table 2:** Hematological parameters of tested horses before and after the training season.

Parameter	Before the season	After the season	p-value
WBC (10^9^.l^−1^)	5.997 ± 1.488^A^	8.002 ± 2.781^A^	*^A^
LYM (10^9^.l^−1^)	2.287 ± 0.8539	2.079 ± 0.3889	ns
MID (10^9^.l^−1^)	0.179 ± 0.188	0.2207 ± 0.201	ns
GRA (10^9^.l^−1^)	3.530 ± 1.153^A^	5.701 ± 2.538^A^	**^A^
LY% (%)	38.62 ± 11.31^A^	29.04 ± 10.95^A^	*^A^
MI% (%)	2.938 ± 2.914	2.514 ± 2.211	ns
GR% (%)	58.44 ± 11.15^A^	68.44 ± 10.27^A^	*^A^
RBC (10^12^.l^−1^)	7.228 ± 0.716	7.488 ± 0.867	ns
HGB (g.l^−1^)	144.1 ± 11.56	145.6 ± 16.18	ns
HCT (%)	26.61 ± 2.051	27.55 ± 2.560	ns
MCV (fl)	36.85 ± 1.819	37.00 ± 2.038	ns
MCH (pg)	19.98 ± 1.191	19.48 ± 1.015	ns
MCHC (g.l^−1^)	541.1 ± 14.92	528.0 ± 18.40	ns
RDWc (%)	24.73 ± 0.621	24.58 ± 0.755	ns
PLT (10^9^.l^−1^)	64.85 ± 23.83^A^	106.8 ± 33.68^A^	**^A^
PCT (%)	0.043 ± 0.016^A^	0.071 ± 0.023^A^	**^A^
MPV (fl)	6.477 ± 0.667	6.736 ± 0.506	ns
PDWc (%)	33.43 ± 3.407	35.34 ± 2.039	ns

Legend: *p < 0.05; **p < 0.01; ***p < 0.001; ****p < 0.0001.*Within the same row. means with same letters differ significantly (*P < 0.05; **P < 0.01; ***P < 0.001).

### 3.9 Biochemical parameters and ions

The effects of load can significantly disrupt horses’ internal milieu. In our study the effect of different loads on biochemical parameters of experimental horses were also analyzed (Table 3).

**Table 3:** Biochemical parameters of tested horses before and after the training season.

Parameter	Before the season	After the season	p-value
**Mineral profile**			
Ca (mmol.l^−1^)	3.014 ± 0.218^A^	3.484 ± 0.240^A^	****^A^
P (mmol.l^−1^)	0.836 ± 0.151	0.970 ± 0.282	ns
Mg (mmol.l^−1^)	1.051 ± 0.247	0.978 ± 0.146	ns
Na (mmol.l^−1^)	142.5 ± 2.6^A^	138.8 ± 1.8^A^	***^A^
K (mmol.l^−1^)	3.55 ± 0.476^A^	4.19 ± 0.406^A^	**^A^
Cl^−^ (mmol.l^−1^)	102.9 ± 1.91	101.7 ± 2.23	ns
**Nitrogen profile**			
Urea (mmol.l^−1^)	3.622 ± 0.373^A^	4.334 ± 0.736^A^	**^A^
TP (g.l^−1^)	62.97 ± 4.689	63.54 ± 3.211	ns
**Energy profile**			
Glucose (mmol.l^−1^)	5.922 ± 0.362	5.508 ± 0.942	ns
TAG (mmol.l^−1^)	0.502 ± 0.116	0.430 ± 0.138	ns
**Hepatic profile**			
AST (μkat.l^−1^)	4.572 ± 0.369	5.035 ± 0.684	ns
ALT (μkat.l^−1^)	0.136 ± 0.043^A^	0.273 ± 0.075^A^	****^A^
GGT (μkat.l^−1^)	0.242 ± 0.083	0.313 ± 0.148	ns
ALP (μkat.l^−1^)	3.673 ± 1.049	4.329 ± 1.097	ns
Cholesterol (mmol.l^−1^)	2.043 ± 0.360	2.186 ± 0.205	ns
Bilirubin (mmol.l^−1^)	23.83 ± 4.400	23.72 ± 3.820	ns
Creatinine kinase (μkat.l^−1^)	4.436 ± 1.950	4.601 ± 2.504	ns

Legend: *p < 0.05; **p < 0.01; ***p < 0.001; ****p < 0.0001.*Within the same row. means with same letters differ significantly (*P < 0.05; **P < 0.01; ***P < 0.001).

From our results certain changes in concentrations of calcium, sodium and potassium were detected. The changes can be caused by increased sweating but even through, the values were within the reference range for horses. Therefore, it can be stated that the load did not significantly affect mineral profile of observed horses. Changes in electrolyte concentration in blood show the effort of body to compensate osmolarity of extracellular and intracellular space. There have been certain changes in ion concentrations – loss of these electrolytes during long lasting load cause faster onset of fatigue.

Values of mineral profile shown some statistically significant changes. Concentrations of calcium after the season significantly increased compared to the ones before the season. Sodium concentrations significantly decreased after the season compared to the results obtained before the experiment started and we observed opposite trend with concentrations of potassium.

A significant increase in concentrations of urea after the season compared to the values measured before the season was detected. Also, an increasing trend in concentrations of total proteins even though not statistically significant was detected. Any statistically significant changes in concentrations of energy profile parameters were observed.

In hepatic parameters, increasing trends in all parameters except bilirubin were found. Only alanine aminotransferase shown significant increase in values obtained after the season compared to the results from the start of the season (Table 3).

## 4 Discussion and conclusions

Our results show an increase in saline cortisol concentrations in all types of loads that horses have been subjected and only statistically significant changes were observed in the “transportation” group.

The monitoring of factors affecting cortisol concentrations during the circadian rhythm in horses was studied [23]. Cortisol values during the day in racehorses show a trend with the highest values were between 6 and 9 a.m., then they decreased between 10 a.m. and 1 p.m. and continue to decrease during the day between 2 and 5 p.m. The lowest concentration was detected between 6 and 9 p.m. [24]. The cortisol values measured throughout the competition day increased by approximately 55% in horses compared to the values measured at rest and were highest 20 min after the start of the event (90% increase in saliva cortisol levels). In our experiment also an increase in cortisol levels but only 14% compared to the values at rest was observed. This phenomenon could be caused by the complexity of the competition or better adaptation of animals to stressful conditions.

In other study salivary cortisol concentrations in warm-blooded animals during competitions in Denmark were observed [25]. Specially, they focused on the five-year-old Danish Warmblood competition (year 2013) and the Danish National Stallion competition (year 2014) and monitored cortisol concentrations during dressage and parkour. In the competition in 2013, the concentrations of cortisol at rest reached 1.73 ng ml^−1^ and 1.14 ng ml^−1^ respectively. After the competition (2014), the value also dropped to 0.73 ng ml^−1^. Our measured cortisol concentrations increased in all horses after the race. The effect of training under farm conditions and during sports event on plasma cortisol concentrations was studied [26]. The measured values at rest during the different loads were in the range of 52.3–61.8 ng ml^−1^ during the sports event and 40.3–64.8 ng ml^−1^ during the training on the farm. After the load at the sports competition ranged from 50.5 to 74.1 ng ml^−1^, respectively 55.8–73.8 ng ml^−1^ during training on farm. A similar tendency was detected in study as an increase in cortisol concentration while riding – 3.86 ng ml^−1^ to 4.09 ng ml^−1^ was found. The rate of increase in blood cortisol concentration in the above-mentioned experiment is very comparable to our findings, even though our changes were not statistically significant.

Hormones of athletic horses from Spain were observed during training and competitions in another experiment. Post-training cortisol concentrations were almost twice as high as the pre-training samples in all groups of horses (inexperienced, moderately experienced, experienced). During the competition, the values measured before the competition were significantly higher than those measured at rest before training. There are multiple possible causes of this – for example noise, high concentration of people around the stable and constant movement around the animals. The values measured after competition were around 33% higher than before the competition [27]. In our samples from trainings and competition, lower increase in salivary cortisol concentration was recorded, which could be due to the higher experience with conditions in competitions and/or less stressful environment during competitions.

Concentrations of cortisol in experienced vs. unexperienced horses in Gymkhana competition was observed and increase of total cortisol was found in experienced horses 30 min after the exercise. In inexperienced horses, cortisol concentrations were increased in both time periods after the exercise (5 min and 30 min after competition) [28].

Saliva cortisol was also monitored in Arabian horses during the competition season. Concentration measured during the rest was 0.384 ng ml^−1^. Immediately after the end of the competition the cortisol concentration increased to 1.976 ng ml^−1^ and after half an hour when the animal spent at rest and the cortisol concentration dropped to 1.834 ng ml^−1^ [29]. In our experiment, also an increasing trend in the concentration of salivary cortisol was found. The concentration increased from 3.596 ng ml^−1^ to 4.077 ng ml^−1^. However, after the competition (at rest), the concentration dropped to approximately same level as before (3.533 ng ml^−1^). Differences in concentrations could be caused using different diagnostic kit.

Concentrations of cortisol in horses that were, respectively have not been transported to competitions were monitored [30]. In both groups of horses, approximately the same increase in concentration 5 min after the competition, compared to the control sample (40–50% increase) were recorded. In our study, a significant increase was observed change (11.4%). Horses transported for 60 min were salivated immediately after transport, and the cortisol concentration reached 4.1 ng ml^−1^ [31]. The value is almost identical to our measured values during transport, which lasted for 60 min, and our measured values reached 4.16 ng ml^−1^. During 1-h lasting transport, we did not stop the transporter to take samples, therefore the values during transport itself could possibly be even higher. Our data prove that the horse transport stimulates the excretion of cortisol. The same results have been observed in similar studies [[32], [33], [34], [35]].

Concentrations of cortisol in our experiment were in the same range as in the study aimed to observe horse loading before transportation [35], and isolation of horses [[33], [34], [35]]. Compared to plasma cortisol concentrations in other studies [33,34,[36], [37], [38], [39], [40], [41], [42], [43], [44]], the measured values are significantly lower. Only free (unbound) cortisol is present in saliva, but blood plasma contains both free and protein-bound cortisol [45,46]. Total plasma cortisol concentrations are always higher than in saliva.

Transportation, in general, is a stressor that can cause i.e., reactive salmonellosis in horses [47], increase heart rate [42,43,47], cause the presence of hormonal and plasmatic ascorbic acid and increased cortisol levels [32,42,48], changes characteristic of acute stress or cause dehydration [32,42,48]. In contrast, short-term transport has no effect on embryonic death [40] or on behavior during ovulation or the estrous cycle. No effect on estradiol and luteinizing hormone levels has been reported [41,49,50].

In an experiment high-intensity concentrations after training in humans were monitored [51]. In the experiment, significant differences before and after training in the morning (4.88 ng ml^−1^ vs. 6.60 ng ml^−1^ – 26.1% increase) and in the evening (1.56 ng ml^−1^ vs. 2.34 ng ml^−1^ – 33.4% increase) were measured. In our experiment we also noticed an increase in cortisol concentration in horses before and after the most intense training, but the increase was only 6%.

The hormonal response to stress caused by training and competition was examined [52]. Analysis of the data suggested that lower salivary cortisol levels were measured with respect to rest prior to competition, but no statistically significant differences were found between types of loads. Other study was focused on monitoring stress in horses during lunging and salivary cortisol concentrations [53]. There was an insignificant reduction in salivary cortisol levels compared to pre-lunging values. This statement does not correspond with the values measured during our experiment, during which we observed a statistically insignificant increase in salivary cortisol concentration. The reason for this may be the different length of training, or a higher pace of training.

Later a study was aimed to observe cortisol and ACTH concentrations in plasma of warm-blooded horses during treadmill training [54]. During standard training at 3° climb, they noticed increase in cortisol by about 50% in tested horses and 65% in horses that were not used to treadmill training compared to the measurements at rest. This study does not consent with our study where we observed only about 3% increasing trend in cortisol concentrations after treadmill training [55]. The cortisol concentrations usually spike higher in horses with worse physical condition but return to the normal value is the same in trained and untrained horses [[56], [57], [58]]. Overall values are lower in overtrained horses compared to the values obtained from the horses that are being trained regularly [44,58]. These conflicting data reminds us on the importance of study design and including all testing conditions. intensity of training as well as methods used while obtaining samples.

Other study described differences between results from lunging and riding, stated that horses were easier to control while riding [59]. This lets us state that it is very important to keep same rider-horse pairs over the entire duration of experiments to limit the interferences caused by swaps of riders as these bring multiple complications from the scientific point of view. Concentrations of cortisol and other stress indicators in war veterans during therapeutical riding programs were observed [60]. Cortisol concentrations were observed in blood serums. The concentrations before the programme reached 33.9 ng ml^−1^; after the programme they dropped to 31.6 ng ml^−1^ and returned to 33.9 ng ml^−1^ after some time after the programme. In our experiment we did not observe cortisol concentrations in riders, but we can state that the tendencies were opposite to the ones in mentioned study.

Plasma adrenocorticotropic hormone (ACTH) and serum cortisol concentrations increase with illness‐associated stress. In a study, ACTH, cortisol, and ACTH/cortisol ratio significantly decreased over time [61]. The hypothalamic-pituitary-adrenal axis response to stress is far from straight forward, particularly with regards to animal welfare. In a study with fifty-nine horses, from three riding centers in Brittany, France the primary findings show that horses whose welfare was clearly compromised had lower levels of both fecal cortisol metabolites (FCM) and plasma cortisol. Authors have also found that evening plasma cortisol levels positively correlated with FCM levels in horses [62].

The aim of another study was to measure concentrations of cortisol, adrenocorticotropic hormone (ACTH), serotonin, adrenaline, and noradrenaline in horses with various diseases and following surgery, to assess the response of the HPA axis and adrenal medulla. A statistically significant differences between all groups and controls were found for cortisol, ACTH, serotonin, and adrenaline concentrations but only in horses with laminitis and acute abdominal syndrome for noradrenaline [63].

Monitoring of haematological parameters at the beginning and end of the season showed that all measured values were within reference range and horses were in good condition. Values measured while analyzing hematological parameters were within reference ranges [64,65] and no significant changes to horses’ health were observed over the course of the experiment.

A study investigated stressful responses during a 6-week training protocol in young Lusitano horses used for dressage. A significant reduction neutrophil counts was detected [66]. Such findings are compatible with the release of leukocytes from the marginal compartment into the circulating pool under the influence of adrenaline, a typical reaction to acute stress [67]. Similarly hematologic parameters in experimental horses in a similar experiment were within reference ranges [68]. In other study mares showed significant decrease in platelet volume after exercise test [69].

Concentration of biochemical profile parameters have shown some changing tendencies, though only changes in ALT, urea and couple mineral profile parameters were statistically significant. All values were within reference range for horses [64,70] and there were no visible changes to the horses’ health. The concentration of calcium in the plasma of one-year-old thoroughbred horses was 3.03 ± 0.23 mmol l^−1^ during grazing-season and 3.14 ± 0.14 mmol l^−1^ during the following stable-, resp. training-season [71] which are similar to or results.

A significant increase in concentrations of urea after the season compared to the values measured before the season were found. In other study the photoperiod-induced variations and the impact of exercise on oxidative stress biomarkers and biomarkers of metabolic alterations in the blood of Shetland pony mares and stallions involved in recreational horseback riding were studied. The seasonal variations in the antioxidant and substrates of energy metabolism in the blood of mares and stallions, depending on exercise capacity, could be an important aspect in the ability of endogenous adaptive mechanisms of animals to react in advance to environmental changes associated with seasons [72]. Other results and well as our previous findings confirm that various blood parameters show no remarkable changes with training [68,73].

In our study, changes in internal milieu (environment) of tested horses in different stress levels of load were analyzed. Also, blood parameters in experimental horses were observed to detect possible changes related to salivary cortisol. The limitation of this study was the number of animals. But on the other hand, all horsed (n = 14) were housed in the same environment (university jockey club) with the same feeding and welfare standards. To sum up our findings, an increase in cortisol concentrations in all samples collected immediately after the end of exercise in comparison with sample collection at rest (before the experiment) were recorded. Statistically significant differences were found only during horse transportation. Differences between the measured values of cortisol in individual time periods in accordance with sample collection, but mostly no statistically significant differences were found. The concentrations of salivary cortisol after the load had rapidly decreasing trend and in samples collected during racing day a drop to values lower than those collected at rest were observed. For a comprehensive evaluation of the results, it can be stated that the individual forms of load have no significant effect on internal milieu (environment) of horses and the horse management is very important factor that can have effect on salivary cortisol values.

## Funding information

The research was financially supported by projects VEGA 1/0698/22, VEGA 1/0392/20 and APVV-21-0168. This study was supported by the Operational Programme Integrated Infrastructure within the project: Sustainable smart farming systems taking into account the future challenges 313011W112. cofinanced by the European Regional Development Fund.

## Author contributions

1 - Conceived and designed the experiments: Martin Massányi, Agnieszka Greń;, Peter Massányi, Marko Halo<a name = "Line_manuscript_98">

2 - Performed the experiments: Martin Massányi, Marko Halo Jr., Eva Mlyneková, Eva Kováčiková, Katarína Tokárová.

3 - Analyzed and interpreted the data: Martin Massányi, Katarína Tokárová, Marko Halo Jr.

4 - Contributed reagents, materials, analysis tools or data: Katarína Tokárová, Agnieszka Greń, Eva Mlyneková, Eva Kováčiková, Marko Halo.

5 - Wrote the paper: Martin Massányi, Peter Massányi, Marko Halo.

## Declaration of competing interest

The authors declare that they have no known competing financial interests or personal relationships that could have appeared to influence the work reported in this paper.
